# The path to great pediatric septic shock outcomes

**DOI:** 10.1186/s13054-018-2147-1

**Published:** 2018-09-22

**Authors:** Stefanie G. Ames, Christopher M. Horvat, Arno Zaritsky, Joseph A. Carcillo

**Affiliations:** 1 Faculty Pavilion, Children’s Hospital of Pittsburgh, Suite 2000, 4400 Penn Ave, Pittsburgh, PA 15241 USA; 2 King’s Daughters Children’s Hospital, Norfolk, VA USA

The EUCLID study group reports remarkably excellent outcome in children with community acquired septic shock. Successful management of pediatric septic shock is challenging because not all children require the same therapies at presentation, and individual patients require changing therapies over time. The pathway to success is not a ‘one size fits all’ approach. Health care systems and practitioners must be both quick and nimble. Quick because untreated bacterial infections can double in pathogen numbers every 28 minutes [1, 2] and unreversed shock induces epithelial cell and organ injury within 30 to 60 min [3]. Nimble because antibiotic and hemodynamic support needs change over time in unpredictable ways.

The likelihood of success increases with a layered systems level approach. First, the hospital must provide practitioners with resources that facilitate use of time-sensitive septic shock recognition trigger tools and a concomitant one hour–three element bundle that 1) attains a blood culture and gives empiric intravenous antibiotics, 2) gives a 20 mL/kg bolus(es) of intravenous isotonic fluid if there is no hepatomegaly or crackles, and 3) gives a peripheral epinephrine infusion if shock is fluid refractory [4]. Next, the hospital must enable practitioners to seamlessly switch from this protocolized approach to a personalized medicine approach which uses multimodal monitoring to direct targeted antibiotics and source control, fluid boluses, and inotrope, vasopressor, inodilator, and hormonal hemodynamic support therapies [4].

Following initial resuscitative studies demonstrating the benefit of fluid resuscitation and antibiotics in the first hour after identification of septic shock [5], individual emergency department protocols were developed using early recognition trigger tools and time-sensitive administration of antibiotics, fluids, and peripheral inotropes that were associated with reduction in length of stay and mortality [6–8]. National efforts including but not limited to Sepsis 6 in the UK and the American Academy of Pediatric-Children’s Health Care Association (AAP-CHA) Pediatric Septic Shock Emergency Department Collaborative in the US implemented these approaches in multiple center quality improvement programs. Mortality rates were observed to decrease from 11 to 3% within one year in the 22 emergency departments in the AAP-CHA septic shock quality improvement project.

In addition to trigger tool-facilitated recognition and administration of antibiotics and a 20 mL/kg fluid bolus(es) within an hour, further administration of antibiotics, fluid, and hemodynamic therapies in the emergency department and intensive care unit were guided by multimodal monitoring using a personalized approach that maximized benefits while reducing risks of applying therapies without regard to individual pathophysiology and response [9]. The recommended personalized medicine goals [4] in the PICU include 1) a sensitive antibiotic, 2) source control, 3) normal capillary refill with cardiac index > 3.3 L/min/m^2^ and < 6.0 L/min/m^2^ and systemic vascular resistance index > 800 dynes/s/m^2^ and < 1200 dynes/s/m^2^, 4) normal mean arterial pressure minus central venous pressure (MAP − CVP) = 55 + 2 × age mm Hg, 5) central venous oxygen saturation (ScVO_2_) > 70%, and 6) even fluid balance after the initial fluid resuscitation period.

Empiric antibiotics and source control differ according to context. In the EUCLID study in Europe, meningococcemia and *Streptococcus pneumoniae* were most common, with the latter being associated with increased morbidity and mortality; however, in the US *Staphylococcus aureus* and methicillin-resistant *S. aureus* in particular are associated with increased mortality. Culture and sensitivity analyses should be attained as infections differ among individuals. When cultures dictate, empiric antibiotics should be narrowed to the most sensitive antibiotic given at a dosage above the minimum inhibitory concentration (MIC) needed to kill the microbial pathogen. Source control is particularly important in the presence of an abscess or necrotic tissue nidus. Suspicion for and surveillance of nosocomial infection is also warranted. Interestingly, improved outcome with use of peripheral epinephrine infusion compared to peripheral dopamine in patients with fluid refractory shock is associated not only with faster shock reversal but also with a significant reduction in nosocomial infection [10].

Children who present with community acquired septic shock are more likely to need inotropic support (epinephrine) for cardiac dysfunction than children who have hospital acquired septic shock who are more likely to require vasopressor support for vascular dysfunction (norepinephrine) [11]. However, individual patients show varying degrees of hypovolemia, maladaptive vasodilation, and cardiac dysfunction requiring personalized hemodynamic management with inotropes, vasopressors, and inodilators. Bedside limited echocardiography can be used to estimate intravascular volume, left ventricular systolic function, and right ventricular end-diastolic volume as a marker of right-heart strain. Doppler ultrasonography of the ascending aorta or pulmonary artery can be used to calculate cardiac output and guide personalized cardiovascular regimens to attain the desired hemodynamic goals of cardiac index > 3.3 and < 6.0 L/min/m^2^ and systemic vascular resistance index > 800 and < 1200 dynes/s/m^2^ [4].

Central venous pressure alone has limited utility for documenting preload since it is not consistently associated with intravascular volume and may be dynamically affected by therapies such as positive pressure ventilation and the effects of diastolic dysfunction. The goal of therapy is not to achieve a CVP target, but rather to increase perfusion pressure (MAP − CVP) to achieve adequate urine output, distal perfusion, and improved tissue oxygenation. Two studies reported improved survival from septic shock when targeting normal MAP − CVP for age and central venous oxygen saturation (ScvO_2_) > 70% [12, 13]. Another study reported improved survival from meningitis when targeting normal cerebral perfusion pressure (MAP − intracranial pressure) [14].

Separate from appropriate personalized fluid resuscitation in the first hour, administration of fluid beyond the polyuria reserve capacity of the kidney is common in children on mechanical ventilation longer than 24 h related in part to receipt of medications diluted in carrier volumes amounting to more than 30 mL/kg/day [15]. Attention to fluid balance through minimizing unnecessary fluids and personalized use of diuretics or continuous renal replacement therapy to achieve even or negative fluid balance 6–24 h following initial resuscitation is suggested.

Mortality from pediatric septic shock is remarkably low when managed in hospitals that facilitate use of an early recognition trigger tool with a three-element bundle followed by a personalized medicine approach (Fig. 1).

**Fig. 1:**
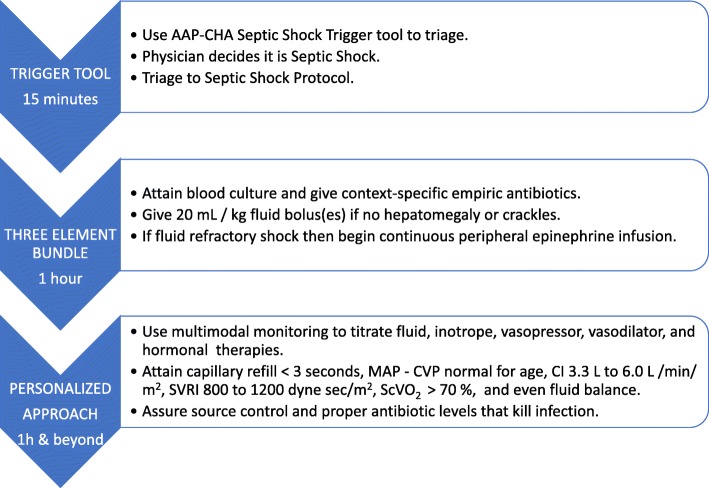
Using an early recognition trigger tool with a three-element bundle followed by personalized medicine to improve pediatric septic shock outcome. *AAP* American Academy of Pediatrics, *CHA* Children’s Health Care Association, *MAP* mean arterial blood pressure, *CVP* central venous blood pressure, *ScvCO*_*2*_ superior vena cava oxygen saturation, *SVRI* systemic vascular resistance index

## Abbreviations

AAP-CHA: American Academy of pediatrics—Children’s Health Care Association
EUCLID: European Community Acquired Sepsis Investigators
MAP – CVP: Mean arterial pressure minus central venous pressure
ScVO_2_: Superior vena cava oxygen saturation
